# Preparation, Drug Releasing Property and Pharmacodynamics of Soy Isoflavone-Loaded Chitosan Microspheres

**DOI:** 10.1371/journal.pone.0079698

**Published:** 2013-11-11

**Authors:** Zhongyan Du, Xiaobing Dou, Chenyun Huang, Jia Gao, Linfeng Hu, Jiazhen Zhu, Ying Qian, Minhua Dou, Chunlei Fan

**Affiliations:** 1 College of Life Science, Zhejiang Chinese Medical University, Hangzhou, PR China; 2 College of Management, Zhejiang Chinese Medical University, Hangzhou, PR China; 3 College of Pharmacy, Zhejiang Chinese Medical University, Hangzhou, PR China; Concordia University Wisconsin, United States of America

## Abstract

Soybean isoflavone (SIF) has anti-aging properties and many other biological functions; however, SIF is difficult to reach higher blood concentration due to its rapid metabolism. Therefore, it is of great value to design and produce a sustained-release formulation that is able to maintain a stable level of plasma concentrations. In this paper, soybean isoflavone sustained-release microsphere from chitosan and sodium alginate was prepared successfully. The important factors that determined the quality of the microspheres were the sodium alginate concentration in solution B, the ratio of soybean isoflavone to chitosan and the mixing speed. The relative yield, encapsulation efficiency and drug loading capability of SIF were much higher than the existing commercial formulations. In real gastrointestinal conditions, compared with the non-sustained release group, the release rate of SIF slowed down and the reaction time was prolonged. Animal experiments showed that sustained-release microspheres intensified the anti-aging potentials of SIF. Compared with the Non-sustained release (NSR) group mice, oral SIF/CHI microsphere treated mice were better in the Morris Water Maze Test (MWMT), the MDA level in the both plasma and brain of the sustained release(SR) group mice decreased, and SOD content was remarkably improved.

## Introduction

Soybean isoflavone (SIF) is a type of flavone compounds, which primarily exists in legumes. It is a secondary metabolite that forms during the growth of soybean. With a chemical structure similar to estrogen, it is also named as a phytoestrogen[1]. Recent research showed that SIF had anti-aging properties and many other biological functions[2] . Ding et al confirmed that SIF had protective effects against inflammations caused by β-amyloid 1-42 (Aβ1-42), which was associated with learning and memory impairment among patients with Alzheimer’s disease[3] .Ma, W et al found that functional deficits in the cognitive capacity of rats in SIF treatment groups were significantly improved compared to the control group (without SIF treatment) in Morris Water Maze Test (MWMT) [3]. In another experiment with 30 ovariectomized (OVX) rats, Lien, T and Hsu Y rats found that SIF significantly enhanced the anti-oxidative activities of the liver extracts[4] . However, the fact that SIF primarily exists in various forms of glycosidic conjugates severely limits its body uptake, as a group of gut microbes are required for its hydrolysis into smaller, more readily absorbable molecules. Consequently, because all SIF products currently sold on the market use an immediate-release mechanism and rapidly dissolve upon their entry into the intestinal tract, flooding the system with excessive amount of the compound that gut microbes are unable to completely process in time, a sustained-release formulation is necessary to improve the clinical value of SIF supplements[5,6]. Therefore, it is of great value to design and produce a sustained-release formulation that is able to maintain a stable level of plasma concentration, giving full play to the biological effects of SIF.

In numerous sustained-release systems, chitosan-alginate microsphere is an ideal slow-release system. Chitosan is the product of chitin after a deacetylation reaction[7] . Chitin is inexpensive and easy to obtain, as it is widely exist in the shell of invertebrates, insect exoskeletons, and the fungal cell wall, making it one of the most common natural resources, second only to cellulose. Research had shown that chitosan had excellent biocompatibility and biodegradability that was wildly used by researchers in the fields of medical, pharmaceutical, food, chemicals, and agriculture[8]. Bio-microspheres, utilizing chitosan as the main raw material, were extensively used for preparations of sustained-release drugs[9] . The chitosan-alginate microsphere-wrapped drug was gradually released in the stomachs or intestines after oral administration. This helped to continue the role of the drug by avoiding toxicity and losses due to the digestive process[10]. Currently, chitosan-alginate microspheres for encapsulation are utilized by a variety of drugs, proteins (peptides), hormonal substances, and nucleic acids. 

 D-gal is widely used for aging model. Yoo, D obtained aging model rats with D-gal in their study of cell proliferation and neuroblast differentiation [11]. MWMT is a classic method to study spatial learning and memory, usually applied with aging model [12]. In addition, Superoxide Dismutase (SOD) and Malondialdehyde (MDA) is a pair of enzymology target which are used to reflect cell oxidative damage in aging model test. Yang, H investigated the effect of Isoflavone Aglycone (IA) on the learning and memory performance of senescence-accelerated mice. Results showed that senescence-accelerated mice treated with IA performed significantly better in the MWMT cognitive test than the no treatment control. The SOD activities were notably higher and MDA concentration was lower[13]. 

In this paper, we introduced the preparation of SIF microsphere, evaluated its characterization from morphology, relative yield, drug-loading capability (DLC), Encapsulation Efficiency(EE), the release in-vitro/vivo and dedicated its anti-aging effect with MWMT by detecting the SOD activity and MDA level in mice plasma and brain.

## Materials and Methods

### Ethics Statement

This study did not involve non-human primates. All experiments described in this study were performed in full accordance with the guidelines for animal experiments released by the National Institute of Animal Health. This study is approved by the Animal Ethic Committee at Zhejiang Chinese Medicine University (ETHICS CODE Permit NO.SCXK (Zhe) 2008-0036).

### Materials

Soy Isoflavones (83.68%; Including Genistin (72.84%), Genistein (0.45%), Daidzin (9.75%), Daidzein (0.24%), Glycitin (0.25%) and Glycitein (0.15%)) was purchased from D&A Bio-tech, Hangzhou, China. Chitosan (83% deacetylation degree, molecular weight: 300–350 kD) was purchased from AKBIO, Jinan, China. Alginate Sodium was purchased from Jiejing Group Corporation, China. SIF commercial capsules (including SIF (50mg), Genistein, Soybean Oligopeptides,Vitamins B,C,D3,E, etc.) were purchased from Baiai Bio-tech, Ha’erbin, China. D-gal was purchased from Sigma-Aldrich, MO, USA. Kits used for the determination of SOD, MDA, glutathione (GSH) and GSH reductase were purchased from Nanjing Jiancheng Institute of Biological Engineering (Nanjing, China) . All chemicals used in this study were reagent grade and all organic solvents were High Pressure Liquid Chromatography (HPLC) grade.

### Animals and Treatments

Female New Zealand white rabbits (weighing 2-2.5 kg) and female ICR mice (6-week-old, 20±2.0g) were used as the animal models for *in vivo* release evaluation and in vivo drug-release experiments, respectively. The rabbits were individually housed and the mice were housed in groups of four per cage in an air-conditioned and light-controlled room at 22 ± 2°C and at 70 ± 5% relative humidity. The animals accessed food and water, ad lib. All animals were healthy and free of observable ocular abnormalities. The local ethics committees for animal experimentations approved all experiments. All surgery was performed under sodium pentobarbital anesthesia, and all efforts were made to minimize suffering.

For the MWMT and pharmacodynamics experiments, after one week adaptation, mice were randomly divided into 4 groups (8 in each group). Groups 2, 3 and 4 of mice received daily subcutaneous injection of D-gal 0.5ml at a dose of 80 mg/kg for 30 days. Mice in groups 3 and 4 received daily SIF commercial capsules (see Materials) named as non-sustained release group (NSR)) and SIF/CHI microspheres (named as sustained release group (SR)), respectively, both containing 75 mg/kg isoflavone in saline (0.9% NaCl) by oral gavage for 30 days. Group 2 of D-gal-treated mice served as model, received 1ml saline (0.9% NaCl) without SIF orally for 30 days. Group 1 that served as vehicle control, received of saline (0.9% NaCl) and 1ml saline (0.9% NaCl) without isoflavone orally for 30 days.

### Preparation of SIF-Loaded Chitosan/Alginate Microspheres

The soy isoflavone-loaded chitosan/alginate sustained-release microspheres (SIF/CHI) were prepared using a patented process (Patent Number: ZL200820082409.2). Briefly: The solution B (containing 1.0% /1.5% /2.0%(w/v) sodium alginate with different soy-isoflavone concentrations (0.01/0.02/0.03/0.04g/ml)) was added drop by drop into solution A (containing 2.0/5.0/10.0(mg/ml) chitosan, 1% (w/v) calcium chloride and 1% (v/v) acetic acid) using a patented machine titled the “centrifuge-pelletizer” (Patent Application Number: 200810060112.0) with different mixing speeds (200/300/400/500 rpm) to form white microspheres with a diameters range 100-200um. 

### Morphology Characterization of Soy Isoflavone Microspheres

The shape, size, sphericity and surface finish quality of all batches of chitosan microspheres were preliminarily checked before drying and after drying by Stereo Microscope (SM, Jiangnan Optical Company, Z00M645, China). The size distributions of various batches of microsphere samples were done on random sampling basis of 100–150 individual microspheres to minimize potential selection bias. The microsphere morphology of chitosan microspheres with emphasis on the surface characteristics and drug-release pore configurations of those microspheres was examined after drying by Scanning Electron Microscopy (SEM, Hitachi, S-3000N, Japan). 

### Relative Yield Evaluation of Soy Isoflavones Microspheres

The microspheres were weighed after drying in 50°C for 24 hrs. Relative yields (RY) (%) of 3 different batches were calculated based on the amount of dried microspheres of each batch obtained relative to the amount of solid materials used in the dispersed phase. The following formula was used:

RY (%) = Amount of dry microsphere / (Alginate Sodium Amount + SIF Amount) ×100% 

### Quantification of Soy Isoflavones

In order to quantify the amount of SIF from microsphere formulations, an improved ultraviolet (UV) spectrophotometry method has been developed. In this method, certain amount of microspheres containing SIF were weighed and disintegrated in 10mL phosphate buffer solution (PBS, pH 7.4), at 37°C, 100 RPM overnight. Then the SIF were completely extracted using chloroform repeatedly 6-8 times until there was no absorbance at 260nm, main absorbance wavelength of SIF. The organic phase liquid was collected and evaporated under nitrogen gas. 2ml ethanol was added over the residue and vibrated for 15 min. Afterwards this mixture was filtered through 0.45mm polytetrafluoroethylene (PTFE) filters for UV detection in 260nm. The method was validated through full wavelength scanning and the parameters of linearity, accuracy, precision, selectivity and system suitability. 

### Determinations of Drug-Loading Capability (DLC) and Encapsulation Efficiency (EE)

The amount of SIF entrapped into the microspheres was determined over 3 different batches using the validated UV spectrophotometry method described above. The Determination of Drug-Loading Capability (DLC) and Encapsulation Efficiency (EE) were analyzed using the following formulas: 

DLC (%) = SIF Amount in Microsphere / Dried Microsphere Total Amount × 100%

EE (%) = SIF Amount in Microsphere / SIF Dosage × 100%

### Determination of *In Vitro* Release (R%)

The *in vitro* release profiles of SIF from microsphere formulations were investigated in 500mL release medium solution (simulated intestine fluid (PBS, pH 1.0), phosphate buffer (pH 3.0), acetate buffer (pH 6.0) or simulated intestine fluid (PBS, pH 7.4)) in the dissolution test system using paddle method described in Chinese Pharmacopoeia (2005 version). Microspheres (50 mg/group) were weighed and suspended in the release medium at 37±5°C. The release profiles of the microsphere formulations were investigated over six different flasks for all formulations. The rotation speed of the paddle was set at 100 rpm. One mL of sample was withdrawn from the release medium and replaced immediately with the fresh medium at different time points (0h, 1h, 2h, 4h, 8h, 12h, and 24h). The samples were prepared and analyzed with the UV Spectrophotometry method described above. The *in vitro* release (R%) of each time point were analyzed using the following formulas: 

$$R\%=\frac{C_{N}\times 500+{\text{m}}_{N-1}}{W}$$

C_N_: SIF concentration in different time

m_N-1:_ Removed SIF Mass

W: SIF Amount in microsphere

### *In Vivo* Release in Animal Model

According to weight, 27 female rabbits were randomly divided into 3 groups: vehicle control group, non-sustained release group (NSR) and sustained release group (SR). The vehicle control group was given empty capsules. The NSR group was given commercial SIF capsules. The SR group was given the same mass of SIF/CHI microspheres packaged in empty capsules as the NSR group. All animals were fed with different formulations according to the weight (50mg/kg) after fasting for 30h. Plasma and urine supernatant were collected at 0h, 6h, 12h, 18h, 24h, and 48h after dosing. The fluid was mixed with anhydrous alcohol. The supernatant was collected, desiccated and reconstituted with ethyl acetate after centrifugation. SIF content in the blood or urine was calculated according the supernatant absorbance at 260nm by UV spectrophotometer. The rabbits of the sustained-release group were dissected 24h after dosing in order to visualize the release and disintegration of the microspheres in their digestive systems.

### Morris Water Maze Test (MWMT)

MWMT was carried out after the mice were treated according to *Animals and Treatments* in *Methods and materials* section. The process of MWMT consisted of 4-day learning, memory training and a test including two trials: place navigation trial and spatial probe trial on day 5[14]. Animals were trained in a circular pool (100 cm in diameter) located in a lit room with visual cues. An escape platform (9 cm in diameter) was submerged 2.0 cm below the surface of the pool water, which was maintained at 23 ± 2°C, and mixed with milk powder to obscure the platform. The location of the platform remained in the center of northwest quadrant throughout the 4-day training period. On each day, the mouse was trained for one trial, which lasted for 90s or ended when the mouse reached the submerged platform, thus escaping from the water maze. Before the first trial, each mouse was put on the platform for 15s, then given a 60s free swim and then assisted to the platform where they remained for another 15s rest. Then the mice were released into the water facing the wall of the pool, in turn of north, south, east and west for each trial. Whether the mice found or failed to find the platform within 90s, a rest on the platform for 30s was conducted. Latencies to escape from the water maze (finding the submerged escape platform) were collected in the place navigation test. The spatial probe trial was made by removing the platform and allowing each mouse to swim freely for 60s inside the pool. The time of swimming for each mouse spent in the target quadrant (where the platform was removed) and the number of times each mouse crossed over the target quadrant were recorded with a computerized video system.

### Preparation of Tissue Samples

Mice were decapitated 60 min after the behavioral tests. The liver tissue and the brain were separated and placed directly on ice. The cerebrum was longitudinally bisected along the axes. For the biochemical estimation, the liver tissue and the left cerebral hemisphere were homogenized with ice-cold saline containing protease inhibitor solution (Sigma-Aldrich, MO, USA) to be 10% (w/v) homogenate. The homogenates were then centrifuged at 12,000 RPM for 10 min at 4°C. The supernatant of homogenate was used in the following assays. For pathological studies, following pre-fixed in 10% methanol for 24h, the right cerebral hemisphere was post fixed in 70% ethanol for at least 12h for histopathological examination according to described methods.

### Assay of Superoxide Dismutase (SOD) Activity, Malondialdehyde (MDA) Level and Glutathione (GSH) Content

SOD activity was detected based on its ability to inhibit the superoxide anion free radical of O2- generated by the xanthine/xanthine oxidase system[15]. The MDA level in brain was determined according to the Thiobarbituric Acid method described by Uchiyama and Mihara[16]. MDA levels were expressed as nmol per mg of protein. Glutathione (GSH) level was measured according to a previously described chemical colorimetric analysis method[17]. GSH levels were expressed as μg per mg protein. The above targets were detected according to the kit instructions.

### Statistical Analysis

Data from the MWMT were analyzed with two-way analysis of variance (ANOVA) followed by the Student-Newman-Keuls test for multiple comparisons among different groups. The other data were analyzed with one-way ANOVA followed by Student-Newman-Keuls test. Results were presented as mean±SD. The acceptable level for statistical significance was 0.05.

## Results

### Morphology Characterization of SIF/CHI Microspheres

**Figure 1:**
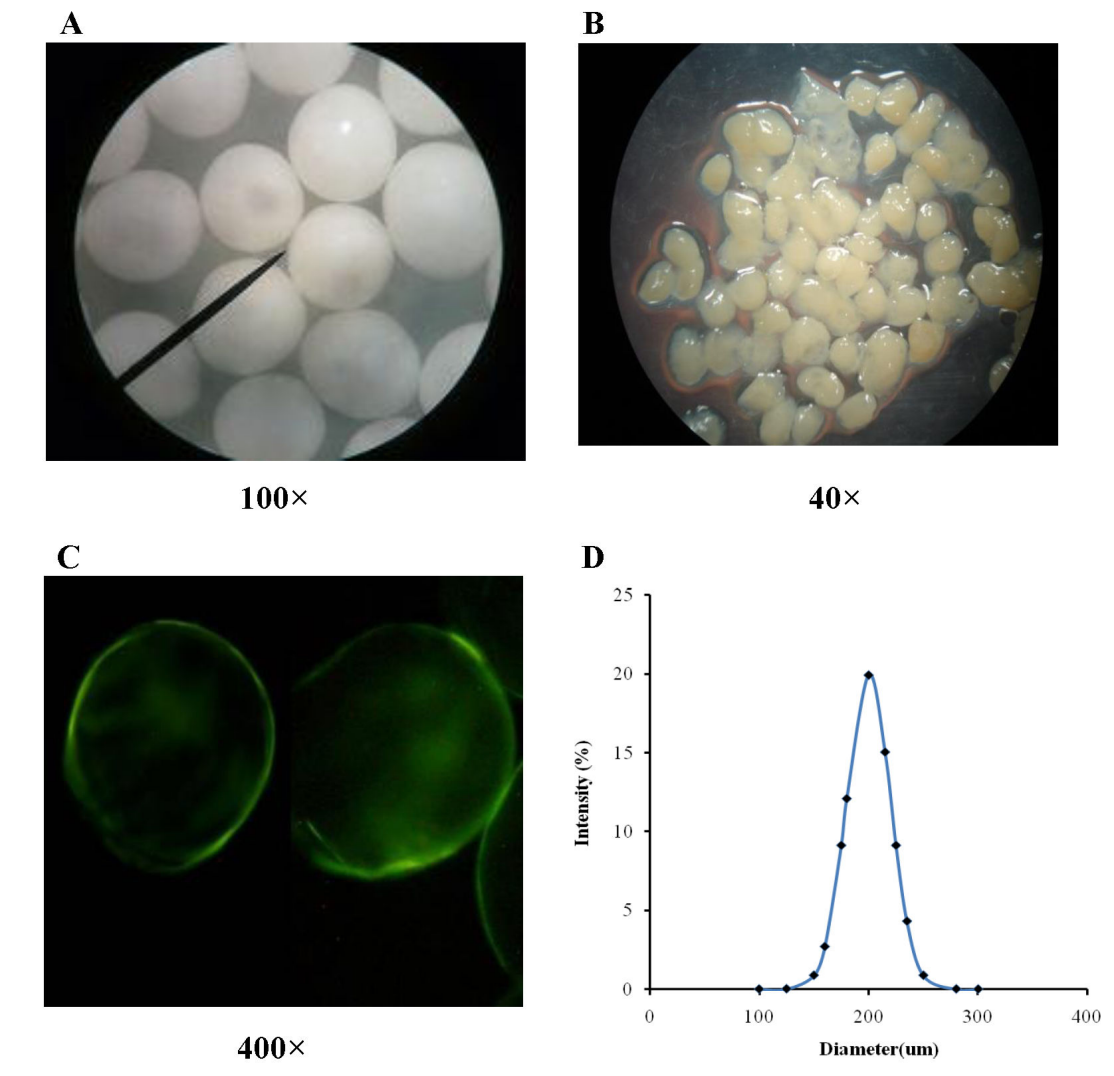
Morphology Characterization of SIF/CHI Microspheres. (**A**-**C**). The surface morphology of microspheres prepared from optimized (A) or unoptimized (B) formulation was investigated by stereo-microscope and by fluoroscopy (C) using FITC-labeled chi-tosan. (**D**). The diameter distribution curve of SIF/CHI microspheres.

Two batches of microspheres were fabricated using two contrasting formulations: optimized (1.5% (w/v) sodium alginate, 0.03 mg/L soy-isoflavone, mixing speed 400 rpm) and unoptimized (1.0% (w/v) sodium alginate, 0.02 mg/L soy-isoflavone, mixing speed 300 rpm). The differently generated microspheres were visualized and compared for their surface morphology using stereomicroscope and fluoroscope as described earlier. The optimized formulation resulted in regular, round microspheres with near-perfect sphericity (Figure 1A and 1C) as well as smooth and homogenous surface texture, while the unoptimized formulation produced irregular ones (Figure 1B). Furthermore, microspheres of optimized formulation showed a near-uniform size distribution around 200µm after drying, with a standard deviation of less than 20µm (Figure 1D). 

### Quantiﬁcation of SIF and RY (%), DLC (%), and EE (%) Evaluation

**Figure 2:**
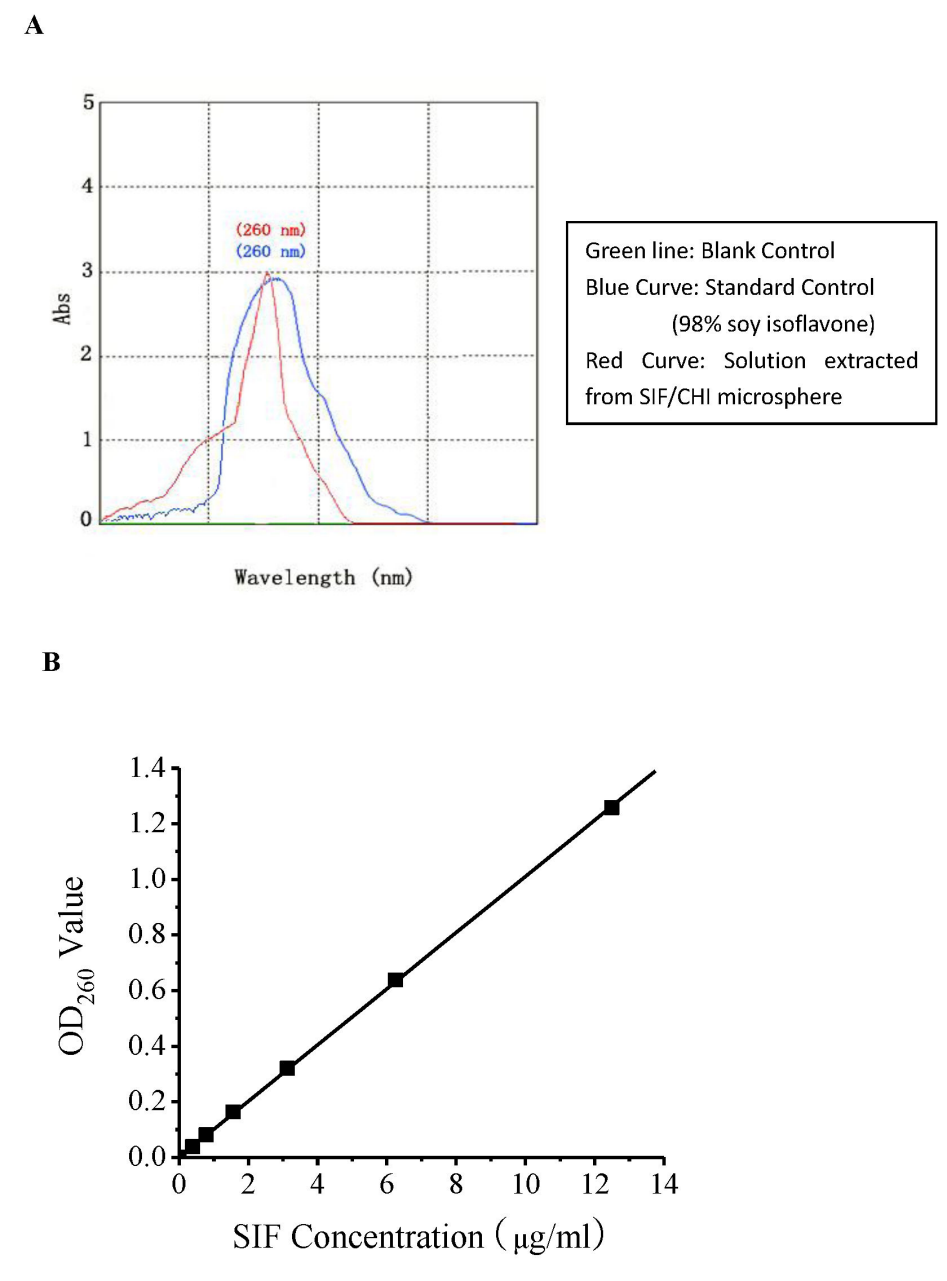
Quantiﬁcation of SIF. (**A**).The UV-visible spectrum of soy isoflavone. (**B**).The standard curve between the absorbance and SIF concentration. Three different batches microspheres were calculated as the formula described in methods. Data are expressed as the mean ± SD.

**Figure 3:**
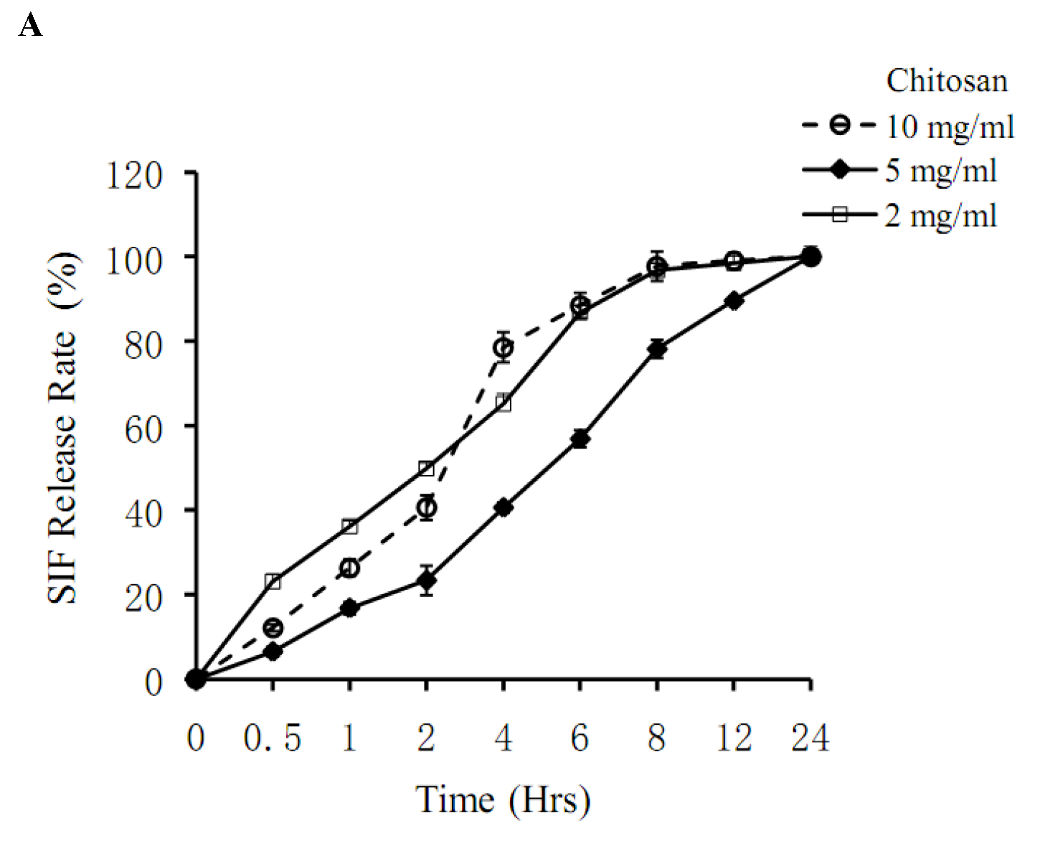
Determination *of In Vitro* Release (R %) of SIF/CHI Microspheres containing different chitosan concentration.

An improved UV Spectrophotometry method was used for determining the concentration of SIF encapsulated in chitosan microspheres. The UV-visible spectrum recorded for a solution of standard sample and dry soy extract solution showed an intense absorption band with a maximum wavelength at 260 nm indicative of SIF (Figure 2A). A similar absorption band was present in the UV-visible spectrum of genistein. Figure 2B showed the standard curve between the absorbance and concentration, which had a linear relationship when the sample concentration was in the range of 0-12.5μg/mL (R = 0.9999). According to the standard curve, Tables 1-3 showed the RY (%), DLC (%), and EE (%) of different batches of microspheres prepared under different conditions. As seen in Figure 3, the concentration of chitosan had a significant impact on the rate at which SIF was released from the microspheres. Among the three concentrations assayed, microspheres consisting of chitosan at 5 mg/mL were shown to be the best for the sustained release of SIF. In comparison, at chitosan concentration of 2 mg/mL, microspheres exhibited the fastest release of SIF among the three at the early stage of the experiment, whereas at 10 mg/mL, an acceleration was observed between T=2 h and T=4 h. As a result, the best microsphere was prepared under the following optimized conditions: solution B containing 1.5% (w/v) sodium alginate, 0.03 mg/L soy-isoflavone, 5 mg/ml chitosan, when mixing speed achieved 400 rpm. All of the SIF/CHI microspheres used in the following experiments were prepared according to the optimized conditions.

**Table 1:** The effect of sodium alginate on the quality control indicators of SIF/CHI microspheres (Mix speed is 400 rpm; SIF addition dosage is 0.03g/ml).

**Alginate concentration**	**Sphericity Surface finish quality**	**Diameter distribution**	**Relative Yield(%)**	**Drug-loading capability(%)**	**Encapsulation efficiency(%)**
1.00%	+	++	79.5	23.6	62.5
**1.50%**	**++**	**++**	**92.4**	**39.4**	**71.3**
2.00%	++	+	98.6	28.8	66.7

The criteria for the assessment: 1) The sphericity and surface finish quality was divided into three levels, namely "+ + + - " to mean " good, fair, poor", using the stereo microscope and optical microscope observation method. 2) Each batch of about 100 microcapsules prepared were observed using the optical microscope. The microsphere particle size distribution was denoted with "+ + + - " to mean "extreme narrow, narrow, wide”.

**Table 2:** The effect of mixing speed on the quality control indicators of SIF/CHI microspheres. (Alginate concentration is 1.5%; SIF addition dosage is 0.03g/ml).

**Mixing Speed(rpm)**	**Sphericity Surface finish quality**	**Diameter distribution**	**Relative Yield(%)**	**Drug-loading capability(%)**	**Encapsulation efficiency(%)**
200	+	+	94.9	21.3	74.7
300	+	++	92.4	32.7	71.9
**400**	**++**	**++**	**91.6**	**38.8**	**70.7**
500	++	+	82.3	24.6	46.7

**Table 3:** The effect of SIF addition dosage on the quality control indicators of SIF/CHI microspheres (Alginate concentration is 1.5%; Mix speed is 400 rpm;).

**SIF addition dosage(g/ml)**	**Sphericity Surface finish quality**	**Diameter distribution**	**Relative Yield(%)**	**Drug-loading capability(%)**	**Encapsulation efficiency(%)**
0.01	++	++	98.9	28.4	70.3
0.02	+	++	97.3	35.3	64.4
**0.03**	**++**	**++**	**96.7**	**43.2**	**73.2**
0.04	+	+	89.6	45.9	42.1

### Determination of *In Vitro* Release (R %)

**Figure 4:**
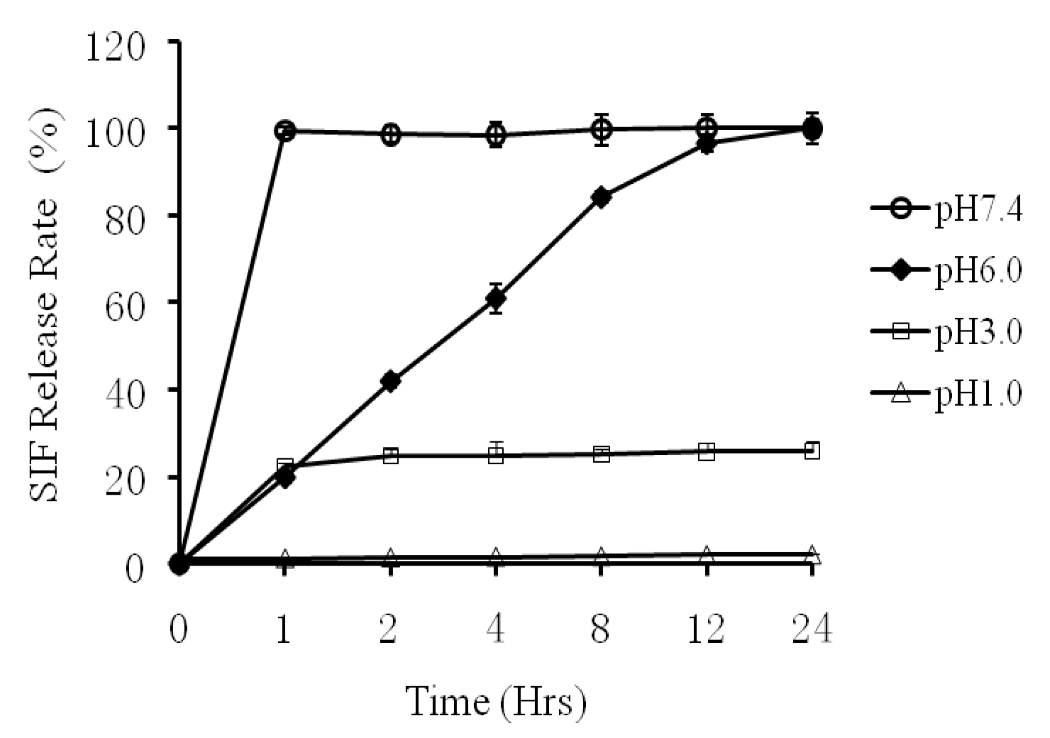
Determination *of* *In* *Vitro* Release (R %). Six samples of different groups of SIF/CHI microspheres were released in different simulation liquids, at 1h, 2h, 4h, 8h, 12h, and 24h to observe the collapse state in each group.

Six samples of different groups of sustained-release microspheres were released in different simulation liquids at 1h, 2h, 4h, 8h, 12h and 24h to observe the collapse of the state in each group. Figure 4 showed that SIF/CHI microspheres placed in simulated gastric (pH 1.0) or single-distilled water, did not demonstrate any significant changes in morphology after releasing for 24h, indicating that disintegration did not occur. However, when the SIF/CHI microspheres were placed in a saline, acetic acid-sodium acetate buffer (pH 3.7), R% was only 20%, even after dissolving for 24h. When the SIF/CHI microspheres were placed in acetic acid-sodium acetate buffer (pH 6.0), R% increased in a time course, achieving a peak of about 100% after dissolving for 24h. Moreover, a fast and complete release of SIF occurred when microspheres were transferred to the simulated intestinal fluid (pH 7.4) after 1h dissolution with the R% was achieved and maintained at 100%.

### Determination of *In Vivo* Release (R %) in Animal Models

**Figure 5:**
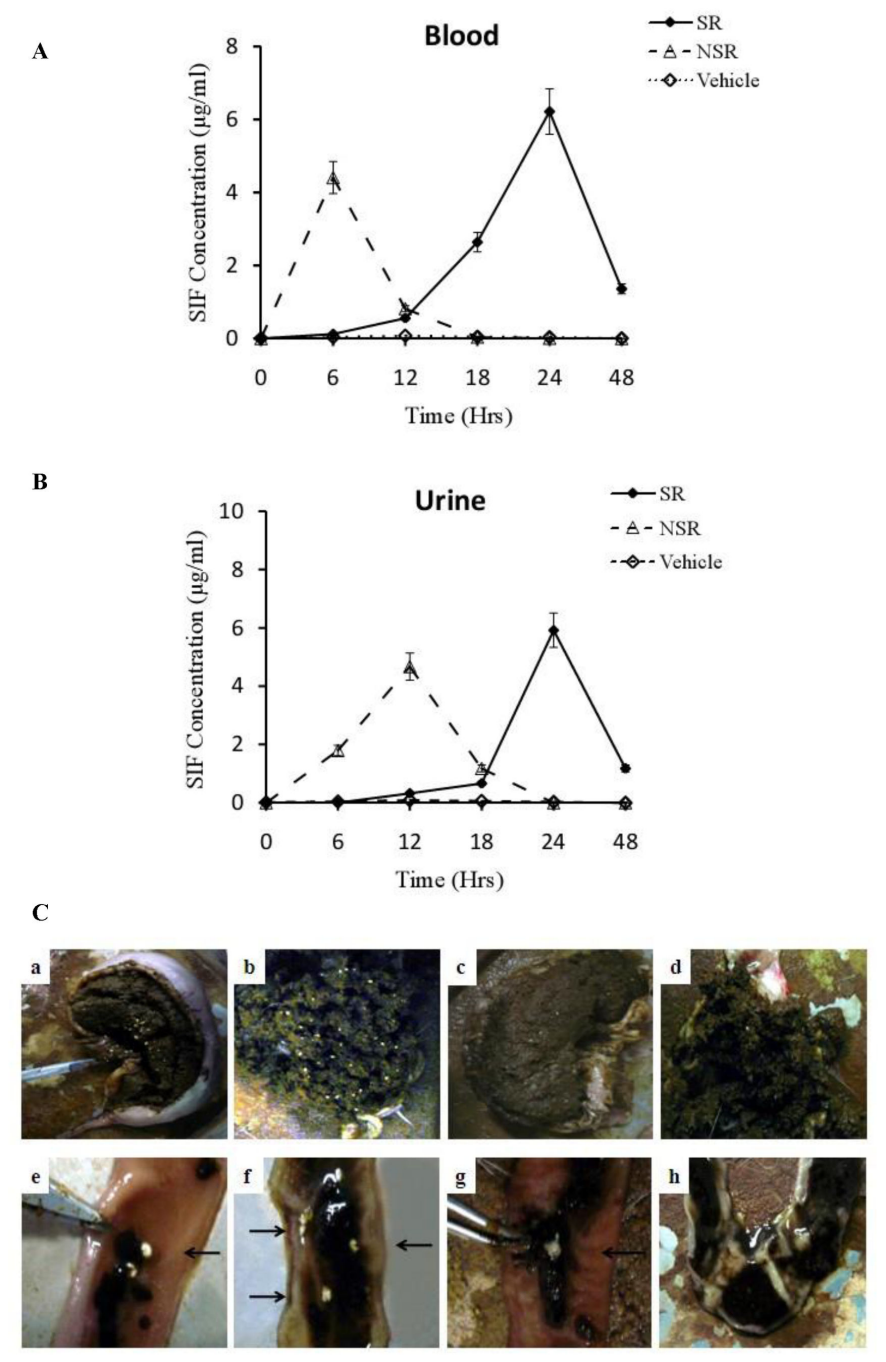
Determination *of* *In* *Vivo* Release (R %) in Animal Models. All rabbits (n=9 per group) were fed with different formulations after fasting 30h. Plasma and urine supernatant were collected at 0h, 6h, 12h, 18h, 24h, and 48h after dosing. SIF content in the blood or urine was extracted and calculated. The microspheres releasing and disintegrating in the digestive system were observed by dissecting the rabbits 24h after dosing. (**A**).The plasma SIF concentrations. (**B**).The urea SIF concentration. (**C**).The disintegration situations in the stomach (a-d) and in the intestine (e-h). All values are denoted as the mean ±SD from at least three independent experiments. Bars with different letters differ significantly (P< 0.05). Vehicle: The vehicle control group rabbits were given empty capsules. NSR: The NSR group rabbits were given commercial SIF capsules. SR: The SR group rabbits were given the same mass of SIF/CHI microspheres packaged in empty capsules as the NSR group.

A typical profile showing the plasma and urine isoflavone concentrations compared to dissolution time and the disintegration situations in gastrointestinal for SIF, after oral administration of the SIF/CHI microsphere capsules in female rabbits, was depicted in Figure 5. Figure 5A plotted the mean plasma isoflavone concentrations for non-sustained release group (NSR) and sustained release group (SR). The plasma SIF concentration in NSR group quickly achieved the peak within 6h after administration and followed by a quick retreat over the next 12h, while the plasma SIF concentration slowly increased, and peaked after 24h in the SR group. The same result was verified in the urine SIF concentration as shown in Figure 5B. Figure 5C showed the disintegration situations in the stomach (Figure 5C, a-d) and in the intestine (Figure 5C, e-h). In the stomach, there were white microspheres 6-12h after administration, while there were little after 12h. The slow disintegration course of microspheres was recorded in images e-h and the microspheres were completely dissolved after 24h.

### Morris Water Maze Test

**Figure 6:**
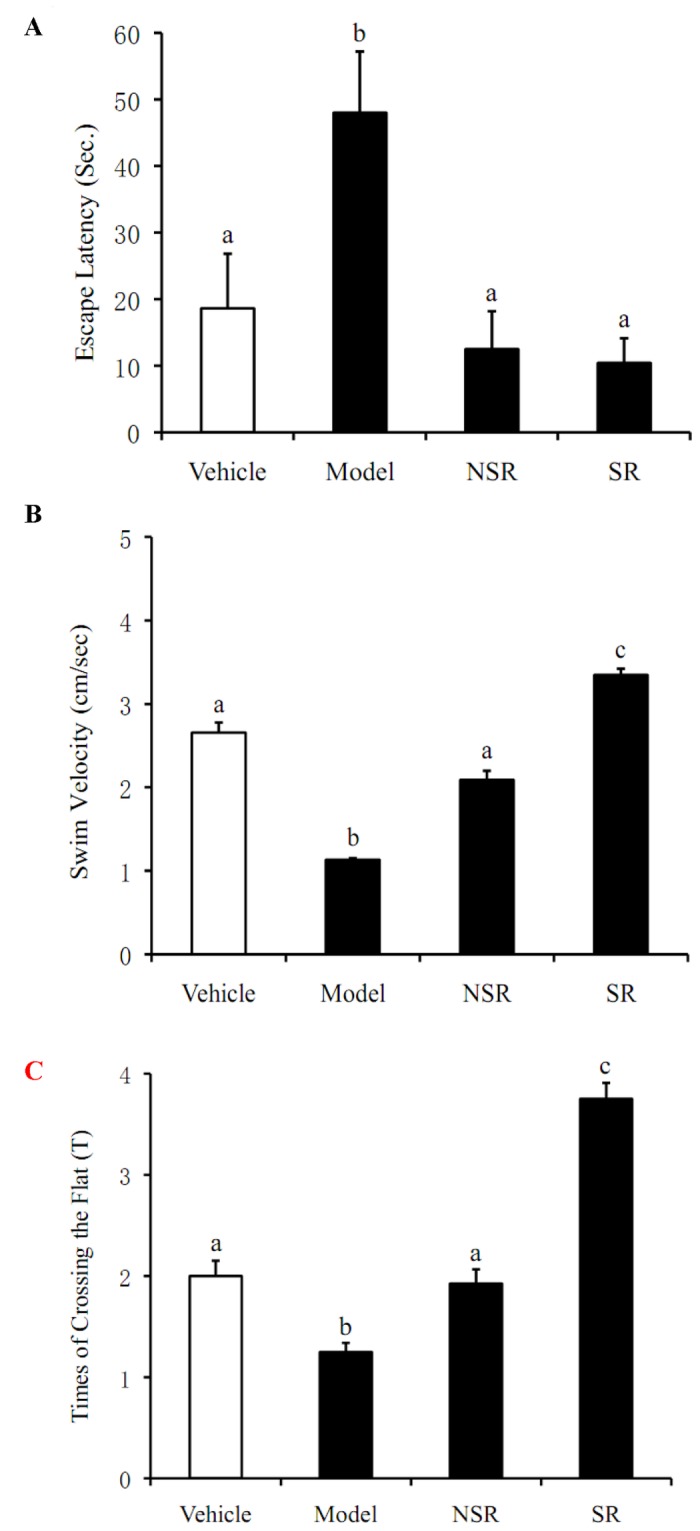
Morris Water Maze Test. All mice (n=8 per group) were trained in a circular pool (100 cm in diameter) located in a lit room with visual cues. An escape platform (9 cm in diameter) was submerged 2.0 cm below the surface of the pool water, which was maintained at 23 ± 2°C, and mixed with milk powder to obscure the platform. The location of the platform remained in the center of northwest quadrant throughout the 4-day training period. Then the mice were released into the water facing the wall of the pool, in turn of north, south, east and west for each trial. (**A**). Latencies to escape from the water maze (finding the submerged escape platform) were collected in the place navigation test. (**B**).The spatial probe trial was made by removing the platform and allowing each mouse to swim freely for 60s inside the pool. (**C**).The time of swimming for each mouse spent in the target quadrant (where the platform was removed) and the number of times for each mouse crossed over the target quadrant were recorded with a computerized video system. All values are denoted as the mean ±SD from at least three independent experiments. Bars with different letters differ significantly from each other (P< 0.05). Vehicle: The vehicle control group mice were given daily subcutaneous injection of saline (0.9% NaCl) and 1ml saline (0.9% NaCl) without isoflavone orally for 30 days. Model: The model group mice were received daily subcutaneous injection of D-gal for 30 days and were given 1ml saline (0.9% NaCl) without SIF orally for 30 days. NSR: The NSR group mice were received the same mass of D-gal and daily SIF commercial capsules containing 75 mg/kg isoflavone in saline (0.9% NaCl) by oral gavage for 30 days. SR: The SR group mice were given the same mass of SIF/CHI microspheres packaged in empty capsules as the NSR group.

 In the present study, the effects of SIF microsphere on learning and memory were examined in mice utilizing MWMT. As shown in Figure 6A, chronic oral administration of D-gal induced significant increase in the escape latency of the mice compared to the control group (*p*<0.05). However, both the commercial immediate-release capsules of SIF (NSR group) and our sustained-release microspheres (SR group) were demonstrated to be capable of restoring the escape latency level back to 10 s, which was statistically identical to the average found in the control group (*p*<0.05). Moreover, the oral administration of sustained-release microspheres were shown to be more effective than the commercial capsules in reversing the adverse effect that D-gal produced on the mice’s swimming and platform-crossing abilities (*p*<0.05).

### Assay of Anti-Oxidant Ability of SIF/CHI Microspheres *In Vivo* Mice

**Figure 7:**
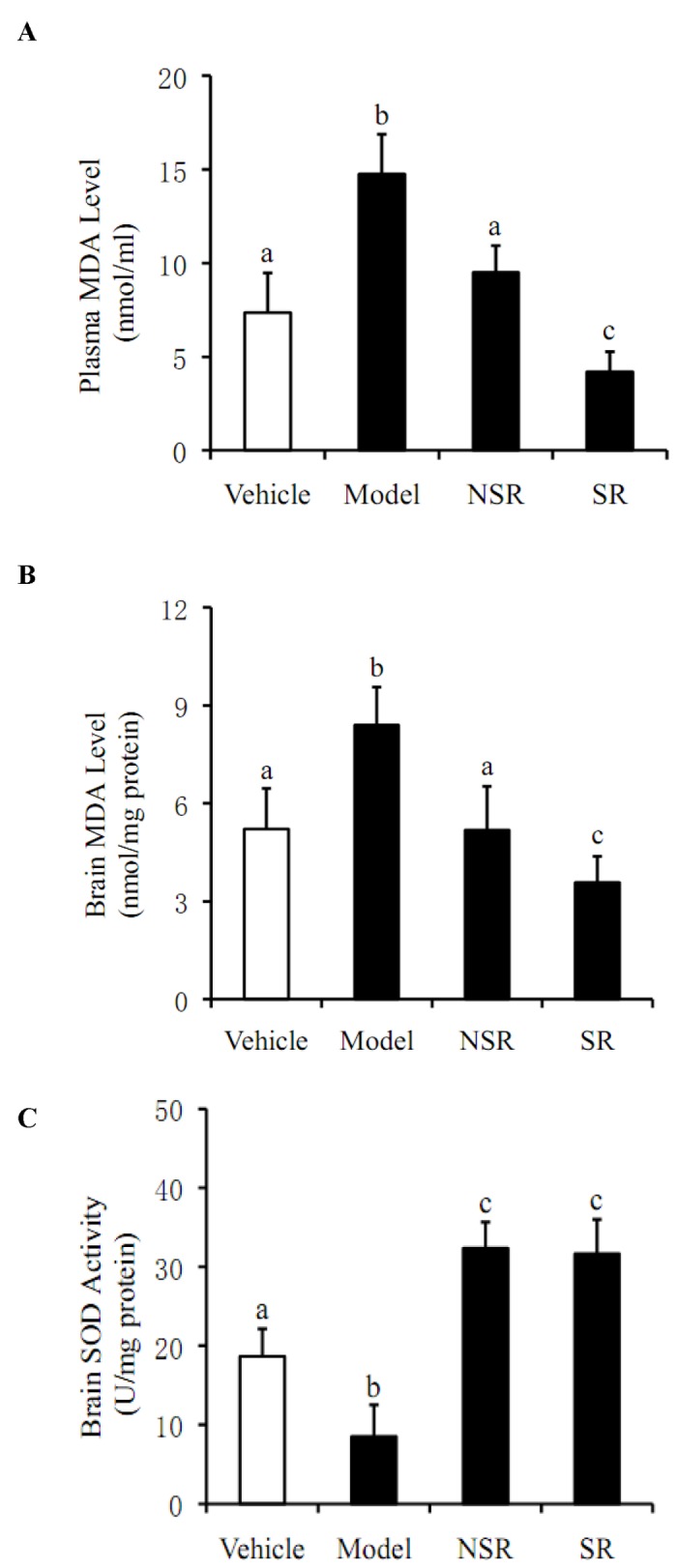
Assay of anti-oxidant ability of SIF/CHI microsphere in vivo mice. All mice (n=8 per group) were decapitated 60 min after the behavioral tests. The plasma and the supernatant of homogenate from the brain were used in the following assays. (**A**).Plasma MDA level analysis. (**B**). Brain MDA level analysis. (**C**). Brain SOD level detection. All values are denoted as the mean ±SD from at least three independent experiments. Bars with different letters differ significantly (P< 0.05). Vehicle: The vehicle control group mice were given daily subcutaneous injection of saline (0.9% NaCl) and 1ml saline (0.9% NaCl) without isoflavone orally for 30 days. Model: The model group mice were received daily subcutaneous injection of D-gal for 30 days and were given 1ml saline (0.9% NaCl) without SIF orally for 30 days. NSR: The NSR group mice were received the same mass of D-gal and daily SIF commercial capsules containing 75 mg/kg isoflavone in saline (0.9% NaCl) by oral gavage for 30 days. SR: The SR group mice were given the same mass of SIF/CHI microspheres packaged in empty capsules as the NSR group.

As shown in Figure 7A and 7B, plasma and brain MDA contents in the model group were higher than those in the vehicle control group (*p*<0.05). However, MDA levels were significantly decreased in NSR group and SR group compared to the model group (*p*<0.05). More importantly, there was a significant difference in the decrease of MDA level between the NSR group and SR group. Figure 7C showed the levels of brain SOD were sharply lower in the model mice than in the vehicle control group (*p*<0.05). Similarly, SOD activity significantly increased in NSR group and SR group compared with the model group (*p*<0.05). However, the SOD activity in NSR group was not significantly different from the SR group (*p*<0.05).

## Discussion

Soybean isoflavone is one of secondary metabolites found in the soybean and has attracted a great deal of extensive attentions in recent years[18], due to its biological functions regarding anti-aging, anti-cancer, anti-osteoporosis and estrogen-like effects. Due to its rapid metabolism when taken directly, it was difficult to achieve a high blood concentration, resulting in a lack of full efficacy[19].Because of this, it is valuable to design a type of SIF sustained-release dosage which can maintain a stable blood concentration level.

This paper described in detail the method of preparation of soybean isoflavone sustained-release microsphere from chitosan and alginate sodium. The method was easy and convenient to conduct and had little restrictions for preparation conditions. This resulted in ease of mass productions, due to the short production cycle, low-cost and high yield. This paper explained that the sodium alginate concentration in solution B, the ratio of soybean isoflavone to chitosan and the mixing speed were the important factors that detemined the quantity of the microspheres. Under the optimized conditions, SIF/CHI microspheres prepared from this process were uniformed in color, and full in shape. The microspheres had good sphericity and finish, fully in line with the morphological evaluation criteria of drug microspheres and with little damages or deformities. For the effect of anti-aging, anticancer and estrogen, isoflavones are being applied to many health care products even medications. However, these products were mostly tablets or soft capsules[20,21,22] with few sustained-release functions. Due to the physical and chemical properties of soybean isoflavones, the existent dosages from these tablets or capsules result in limited blood levels, and thus limit biological effects. Although there are few reports of SIF slow-released microspheres research available, the dosages being used are always prepared by a emulsification or pellet technique to prepared, which is complex, expensive and difficult for large scale productions[23,24]. The method introduced in this paper was more efficient than existing methods to produce slow-release microspheres, and the relative yield was high. In optical microscope observations, these microspheres had uniform sizes, with a smooth surface finish and standard morphology evaluations. 

The qualification standard of enteric slow-release preparation is as followed: the dissolution rate in the acid environment should be no more than 10% and the release rate in the simulated intestinal juice (pH 7.4) must be greater than 70% [25]. Figure 4 demonstrated a release rate of almost 0 in the simulated gastric juice (pH 1.0) 24h after administration, while the dissolution rate increased up to 100% 4 hours after administration in the simulated intestinal juice (pH 7.4). SIF release accelerated at higher pH in the solution. Under conditions that simulated microenvironment inside intestinal tract (pH 7.4), the microcapsules could release more than 99% of the soybean isoflavone after 4h of administration, far beyond the threshold of 70% required for clinical relevancy. Similar correlation between release speed and pH was observed in vivo as shown in Figure 5. The release of SIF remained at minimal level for the first 5-6 h after the administration of the microspheres, which roughly matched the time required for the latter to pass through the acidic gastric environment. Upon entry into the slightly basic intestine, however, the microspheres started to dissolve at an accelerating speed, leading to a rapid increase of blood SIF level from T=6 h through T=24 h. In contrast, the release profile for the NSR group denoted much earlier peaking of SIF in blood and a concomitantly lower peak concentration as a result of insufficient absorption. The above findings strongly supported the advantages of the sustained-release preparation of soybean isoflavone described in this paper, which made sure that the soybean isoflavone was released slowly in the intestine, meanwhile avoiding stomach digestive damage, and solving the existing rapid metabolism of soybean isoflavone agents[26]. In this paper, the absorbance of SIF was determined by UV spectrophotometry, showing a high encapsulation efficiency and drug loading capability of SIF which was much higher than the existing commercial formulations.

In real gastric conditions, slow-release microspheres kept their structure, but dilation and disintegration became much easier once those microspheres entered the intestines through the digestive processes. Fragments of the microspheres were found in the small intestine at 18h after administration, and disappeared ultimately. Compared with the blood concentration which decreased sharply at 6h in NMS group mice, the blood concentration reached the highest level slowly and maintained it persistently. SIF concentration in the plasma and urine were determined with UV spectrophotometry. Compared with the non-sustained release group, the decomposition metabolism of SIF *in vivo* slowed down and the reaction time was prolonged, which was beneficial to the full play of SIF biological efficacies.

The Morris Water Maze Test has been regarded as one of the most frequently used laboratory tools in spatial learning and memory of neurobiology and neuropharmacology research, typically consisting of a series of spatial learning acquisition training, and spatial accuracy memory [27]. Compared with a vehicle control group, behavioral impairment was demonstrated in the model group. Significantly prolonged escape latency, swimming velocity and decreased target quadrant search time were found at D-gal injected mice. However, marked improvement of the behavioral impairment was found in both NSR and SR groups. Oral SIF dose mitigated cell oxidation caused by the D-gal [28].It was remarkable that oral SIF/CHI microsphere treated mice were better in the MWMT, while NSR group mice needed more time to find the target platform while having weaker performance in the space exploration.

As one of the final product of lipid peroxidation, Malondialdehyde (MDA) reflects the level of free radicals directly. Its concentration is an important symbol of cell damage degree. It was found to increase in the ovariectomized rats by Cai Shaofen [29]. Superoxide dismutase (SOD) is an important enzyme in the oxygen free radicals removal, with its content level reflecting the oxidation resistance present [30]. The cytotoxicity of D-gal caused significant decrease of SOD but increase in MDA. Similar results were observed in the model groups of our experiment (not clear). These results were also in consistent with the experiments conducted by Cakatay, U and Aydin, S (et al) in aged rats experiments[31], SIF reduced these specific types of damage, especially with the SIF/CHI microspheres. Compared with the NSR group mice, the MDA level in the both plasma and brain of the SR group mice decreased, and SOD content was remarkably improved in those orally administered SIF/CHI microspheres mice. These animal experiments showed that sustained-release microspheres can intensify the anti-aging potentials of SIF.

## Conclusions

To sum up, the SIF/CHI microspheres had a simple preparation technique and they are stable in nature. They had perfect enteric absorption and sustained releasing effect. Through the animal behaviors and enzymology studies, it was found that compared with the current soybean isoflavone dosages, the slow release microspheres adequately strengthened the SIF anti-aging effect. However, hippocampal nerve cells atrophy and a series of gene expression abnormalities always appeared with aging[32,33]. Can these microspheres alleviate degenerative changes of nervous system and reduce gene abnormalities? Further research is needed. The study of SIF/CHI microspheres is just in the animal experiment stage. Their metabolism and biological efficacy in human still need further investigation. 
